# The History of Epilepsy: From Ancient Mystery to Modern Misconception

**DOI:** 10.7759/cureus.13953

**Published:** 2021-03-17

**Authors:** Christian M Kaculini, Amelia J Tate-Looney, Ali Seifi

**Affiliations:** 1 Department of Neurosurgery, University of Texas Health Science Center at San Antonio, San Antonio, USA; 2 Department of Microbiology, Immunology and Molecular Genetics, University of Texas Health Science Center at San Antonio, San Antonio, USA

**Keywords:** epilepsy, historical medicine, social stigma, seizure, babylonia, ancient greece

## Abstract

Epilepsy is an ancient disease, which has fascinated and frightened scientists and laymen alike. Before the working knowledge of the central nervous system, seizures were shrouded in mystery. In antiquity, this disease was accredited to gods and demonic possession, causing those with epilepsy to be feared and isolated. Epilepsy patients continued to face discrimination through the mid-20th century. This discrimination ranged from lack of access to health insurance, jobs, and marriage equality to forced sterilizations. Despite the strides that have been made, there are still many misconceptions globally regarding epilepsy. Studies show that patients with epilepsy in communities that understand the pathology and cause of seizures are generally more successful in social and educational environments. While there has been progress, there is more work which needs to be done to educate people across the globe about the pathology of epilepsy.

## Introduction and background

Epilepsy as defined by the International League Against Epilepsy (ILAE) is a disease of the brain that results in at least two unprovoked seizures at least 24 hours apart. A person may also be diagnosed with epilepsy if they have one unprovoked seizure and have a high chance (>60%) of having another seizure within the next 10 years or if they have an epilepsy syndrome [1]. Epilepsy is a disease historically associated with evil spirits and mystery, and still to this day often carries social stigmas [2]. Its long history, along with its social implications, makes epilepsy a unique disorder. This review will discuss epilepsy’s extensive history as well as how societal perceptions of people with epilepsy have evolved over time.

## Review

Ancient history

**Figure 1:**
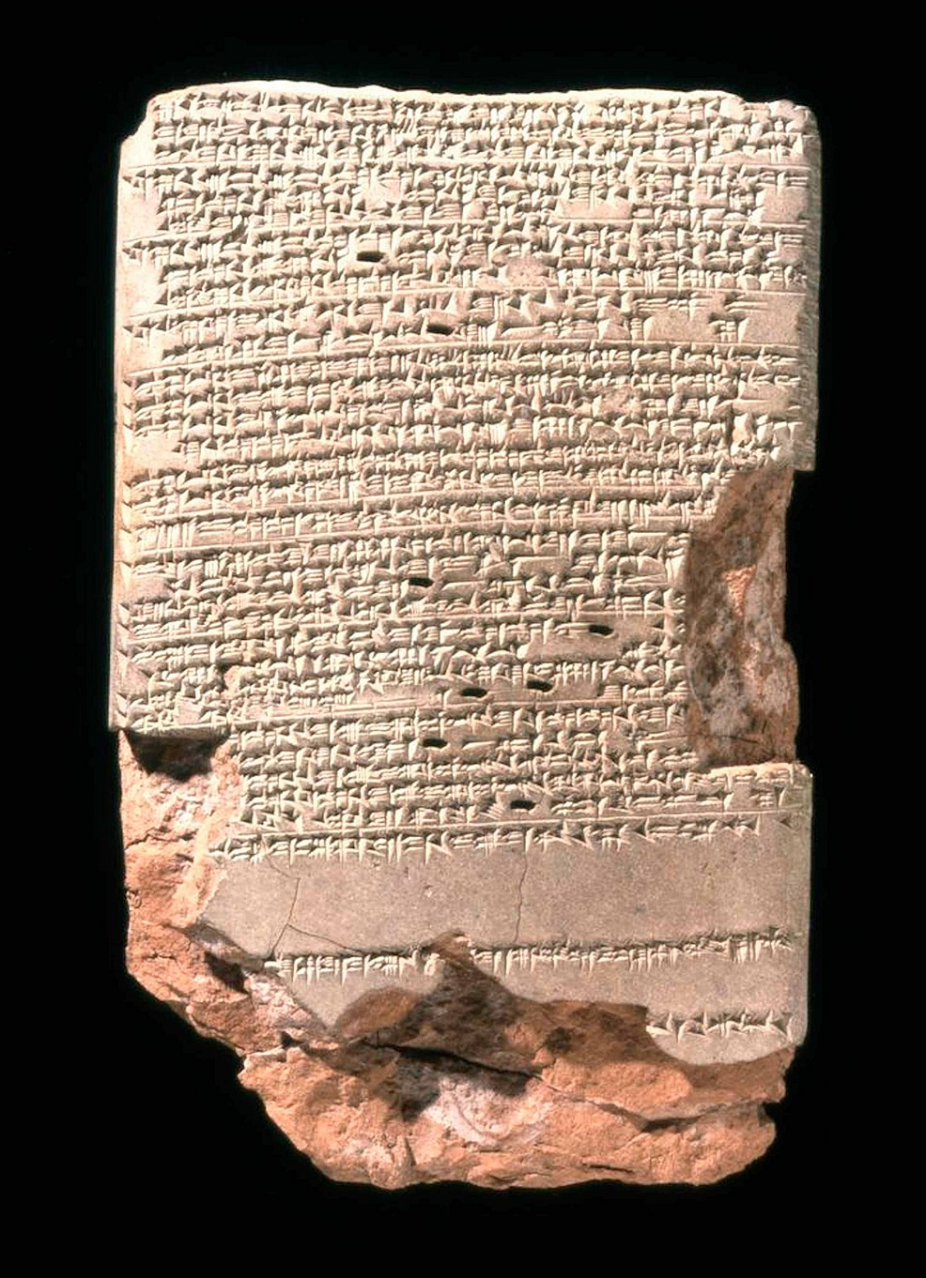
Tablet 26 of a series of 40 which compose the ancient Babylonian diagnostic manual entitled Sakikku which translates to "all diseases". Sakikku is organized into six chapters and tablet 26 is the first in the chapter about epilepsy. It describes the symptoms of epilepsy and the different types of epileptic presentations [5]. Tablet BM47753. Neo-Babylonian Period. Courtesy of the British Museum, London.

Epilepsy's long history can be traced back to a 4000-year-old Akkadian tablet found in Mesopotamia; inscribed on it is a description of a person with "his neck turning left, hands and feet are tense, and his eyes wide open, and from his mouth froth is flowing without him having any consciousness" [2]. Nearly a millennium later, the Late Babylonians wrote a diagnostic manual entitled, Sakikku, which included texts describing epilepsy (Figure 1). In this guide, the Babylonians describe several seizure types and categorized them based on their presentation. They also had some understanding of prognostics, as the text detailed different outcomes depending on the type of seizure, including poor outcomes in status epilepticus, as well as post-ictal states in other seizure types. This tablet further described terms relating to epilepsy such as miqtu (fall), hayyatu (fit), and sibtu (seizure) [3]. This rudimentary nomenclature further underlines that the ancient world had some understanding of epilepsy. Due to the belief that these episodes of rapid contractions were caused by evil spirits invading the body, the treatment often involved spiritual intervention [4].

Evidence of epilepsy has also been found in ancient Egypt, as indicated by the Edwin Smith Surgical Papyrus written circa 1700 BC. It describes several accounts of epilepsy, one of which is of particular interest. The Egyptians documented a case in which direct stimulation of the brain resulted in a physiologic response. The case described a man with "a gaping wound in his head" and when the wound was palpated, the man would "shudder exceedingly" [6]. Distinguishing themselves from the Mesopotamians, who believed spirits and gods were the cause of seizures, the Egyptians proved that seizures can be caused by cortical disruption. Documentation of epilepsy is also found in Chinese texts, dating to approximately 770-221 B.C. A group of physicians published The Yellow Emperor’s Classic of Internal Medicine, Huang Di Nei Ching, which outlined generalized seizures. In 610 A.D, Cao Yuan Fang was thought to have classified and categorized epilepsy. Traditional principles of Yin Yang Wu Xing were employed to treat epilepsy, consisting of herbs, massage, and acupuncture [7].

Epilepsy's spiritually based pathophysiology remained largely unchallenged until around the 5th century BC, when the School of Hippocrates in Greece hypothesized that the brain might be the root cause of epilepsy. Hippocrates believed that the Sacred Disease (epilepsy), was no more divine than other diseases, but was named "sacred" due to its unique and inexplicable characteristic appearance. He also hypothesized that epilepsy could be cured like other diseases, though once it becomes chronic, it was no longer curable [6]. Hippocrates was also one of the first to introduce the concept of post-traumatic epilepsy; through his observations of head trauma, he observed convulsions which were always contralateral to the head wound [6].

In essence, Hippocrates was among the earliest to attribute epilepsy to the brain and to suggest that it is hereditary rather than contagious. He described its clinical presentation as unilateral motor signs with an aura, which could serve as a warning signal that allowed them to immediately leave the public to convulse. During this time, it was widely accepted that epilepsy was caused by spirits, which played a role in the social stigma surrounding epilepsy [6]. He attributed society's misunderstanding and reaction to epilepsy as a result of divine fear which society had built around this disease [6]. Hippocrates was one of the first to explain a non-spiritual basis for epilepsy, but unfortunately, his hypothesis had little influence over the supernatural belief for many centuries to come.

This stigma surrounding seizures, and the misunderstanding of their origins, caused noteworthy impacts on society’s view of epilepsy throughout history. Aristotle, a notable philosopher of the 4th century BC, hypothesized that epilepsy and sleep were due to similar mechanisms. In his work, On Sleeping and Waking, he theorized that sleep was caused by the evaporations resulting from consuming food, which would subsequently rise and fall in the veins. He extended this hypothesis to the process occurring when one convulsed and thought this was the mechanism that caused epilepsy to affect levels of consciousness [8]. In later years, his ideas were considered indisputable by the Catholic Church and would influence the scientific community for centuries. Even famous physicians like Galen included Aristotle's ideas of vapors in his works [6].

The Hippocratic idea that epilepsy was a brain disorder finally began to gain traction in Europe beginning in the 17th century and continuing through the millennium [4]. Samuel Tissot (1728-1797), a prevalent Swiss physician, published Traité de l’épilepsie in 1770 [9]. A decade later he published a four-volume text entitled Traite des Nerfs et du leurs Maladies, which cemented him as a prominent medical figure in the enlightenment period. William Cullen (1710-1790), a Scottish physician, outlined the fact that seizures could occur in parts of the body, and did not inherently have to result in loss of consciousness [10,11]. During the same era, French physician Maisonneuve (1745-1826) began emphasizing the need to hospitalize patients with epilepsy [12,13]

In 1849, Dr. Robert Bentley Todd introduced the idea that the brain functions through an electrical force and hypothesized that “electrical discharges” in the brain may be the cause of seizures [4,14]. He later confirmed his hypothesis using Michael Faraday's magnetoelectric rotation machine on rabbits [15]. John Hughlings Jackson (1835-1911) laid the scientific foundation for epileptology, as well as studied the localization of lesions which could produce seizures [12,16]. He published the influential text, “Study of Convulsion” which was the culmination of his scientific findings. Around 80 years later, Hans Berger invented the human electroencephalogram, which allowed him to confirm that convulsions were the result of abnormal electrical activity in the brain [4]. In 1935, William Lenox demonstrated that there was no change in cerebral blood flow in patients during a seizure, finally dismantling the pervasive belief of a vascular etiology for epilepsy. He also demonstrated abnormal electrical changes before convulsions which increased during a seizure, which he proposed as the new etiology for epilepsy [17]. 

Societal perception of epilepsy

**Figure 2:**
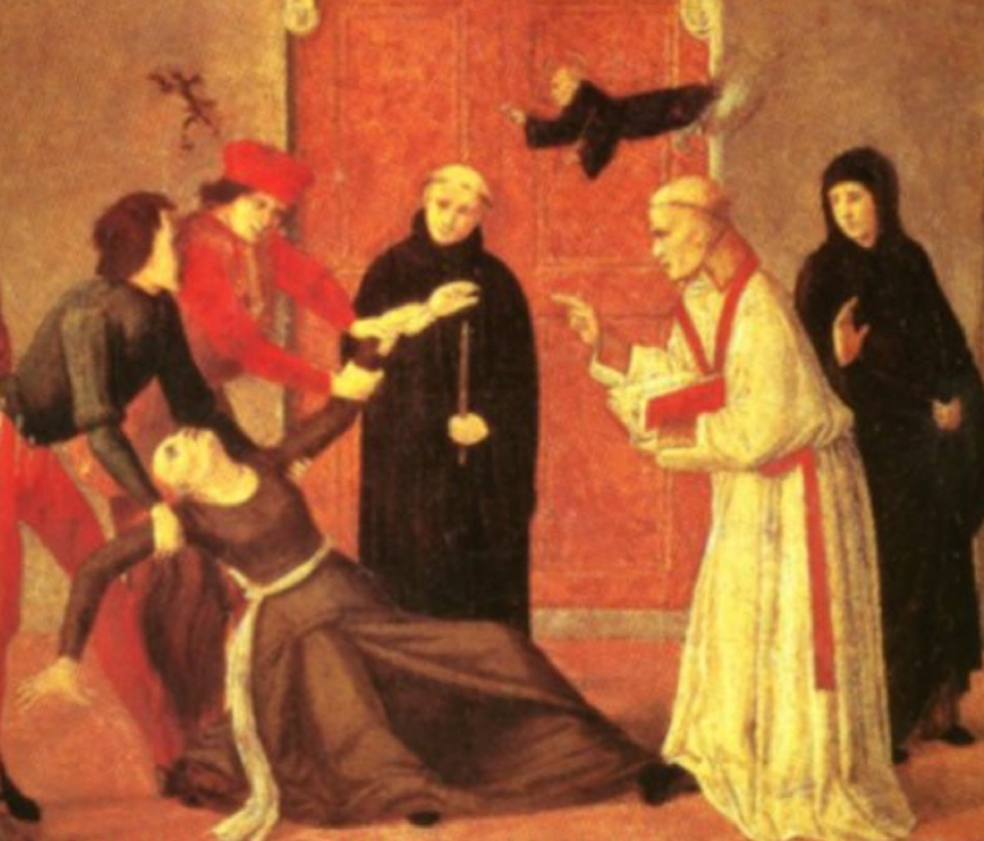
Saint Severin curing a woman of the 'falling sickness demon'. Meister des Heiligen Severin. Circa 1300. Courtesy of the Museum Horne Foundation, Florence.

Clinically, the presentation of a seizure can be sudden and dramatic, which may elicit fear in people. The mystery behind the cause of seizures has been debated for millennia, and many theories and misconceptions have led to profound social consequences for people with epilepsy. Throughout most of history, seizures were thought to be caused by evil spirits invading the body, which required exorcism or other religious and spiritual remedies (Figure 2) [4].

From ancient to relatively modern times, people with epilepsy have been disenfranchised and the subject of discrimination. Until the mid 20th century, in the United States of America, many states prohibited people with epilepsy to get married, and some even encouraged eugenic sterilization [4,18]. Many public facilities, including restaurants, had the right to deny service to people with epilepsy until the 1970s [19]. These discriminatory laws further stigmatized people with epilepsy. Even in recent years, many developing countries continue to perceive epilepsy to be a result of evil or ancestral spirits. In these areas, it is common for the patient and their family to usually see a traditional healer first and follow their treatment recommendations. Epileptic patients often face stigma, which may discourage them from seeking the treatment they need [4]. In some countries, a patient can have symptomatic epilepsy for 6-14 years before seeking modern medical care [4]. Finally, in the late 20th century, several international societies were formed to promote both the scientific and social knowledge of epilepsy. In 1997 the International League Against Epilepsy, the International Bureau of Epilepsy, and the World Health Organization focused their objectives on addressing political and public awareness of epilepsy to reduce stigma and to improve treatment [4].

Modern day patients with epilepsy often face civil rights violations. One example is the unequal access to health and life insurance they may encounter. Another is how people with epilepsy often are discriminated against by employers, frequently causing them to choose a different occupation altogether. This is often seen in "hands-on" professions, such as firefighters or construction workers, despite the fact that accommodations from employers should be available under the Americans with Disabilities Act [20]. An epilepsy diagnosis can impact employment options and the number of employers which will hire them, even when the individual is fully capable of the job. People with epilepsy are often dissuaded from pursuing certain professions because of the speculated consequences of epilepsy. Several countries in Europe still have job restrictions based on a diagnosis of epilepsy despite many worldwide reports of low accident rates in people with epilepsy [21]. These civil rights violations extend to their autonomy, resulting in limitations in legal agreements, such as marriage, in some countries. Until the passage of the Affordable Care Act, many health insurance companies in the United States could deny coverage based on pre-existing conditions, and in 2005 as many as 36% of people diagnosed with epilepsy were refused one or more types of insurance because of their epilepsy [22].

People with epilepsy can be subjected to social ostracism, both directly and indirectly. There is a growing body of evidence that a strong social support system is directly correlated with well-being [23]. According to the Epilepsy Foundation, parents often felt that their journey after their child’s diagnosis was a challenge to navigate, citing difficulties seeing a specialist, financial strain due to medical care, and lack of opportunities for their children due to challenging behavior [24]. Often, those with epilepsy tend to be more isolated than those without, resulting in diminished well-being. Even as early as childhood, people with epilepsy begin showing signs of social issues, stemming from lack of inclusion [25]. This social isolation after a childhood diagnosis can negatively impact self-esteem and academic performance [4]. An example of how those with epilepsy become unintentionally isolated is how social gatherings require transportation, and in areas where public transportation is limited, patients with epilepsy can be excluded socially due to their ineligibility to obtain a driving license. There is evidence that people with epilepsy suffer from higher rates of depression, anxiety, psychosis, and attention-deficit hyperactivity disorder (ADHD) than the general population, indicating that this disconnect from society can lead to harmful downstream effects [26,27]. According to a longitudinal study by Berg et al. seizure course (akin to severity of seizure examine from childhood to young adulthood) influenced completion of college, employment, and driving, which further impacts employment opportunities [28]. This may cause socioeconomic impact, due to a combination of diminished access to the job market, and in the United States of America, lack of health insurance due to joblessness. Due to increased risk of mental health conditions, compounded with the issues discussed above, those with epilepsy were more likely to have a lower annual income and were more likely to be unemployed [29,30]. The more direct effects of ostracism can be seen in developing countries, where there are commonly held misconceptions that epilepsy is contagious. Some believe contact with saliva or the person during a seizure can cause transmission. This leads to social isolation and further abandonment during a seizure, which increases the likelihood of a seizure-related injury [31].

Societal knowledge and perception of epilepsy have been directly correlated to the successful treatment of epilepsy. Hirfanoglu et al. found a correlation between familial understanding of the disease after their child’s diagnosis and fewer depressive symptoms and the better use of anti-epileptic drugs (AEDs) [32]. A similar effect was observed in urban secondary schools in northern India, where the more knowledgeable students were about epilepsy, the less discrimination students with this condition face [33]. These studies (and beyond) indicate that the more that is known about epilepsy and what causes it, the better patients with the disease are incorporated into society, and outcomes and seizure management improve. In essence, the more that is understood about epilepsy by the public, the better the lives are for people with epilepsy.

Modern treatment of epilepsy

At the turn of the 19th century, pharmacologic treatment of epilepsy began to gain traction. In 1912, Alfred Hauptmann discovered the anticonvulsant properties of phenobarbital, one of the most commonly prescribed medications for epilepsy worldwide today [34]. Numerous AEDs were introduced in the following decades including ethosuximide, carbamazepine, valproate, and several benzodiazepines. Today, AEDs are usually the first-line treatment for epilepsy and selected based on the type of seizure one has as well as the patient’s other pertinent medical history. For seizures that are refractory to AEDs, patients can be offered alternative treatments including trying a ketogenic diet, vagus nerve stimulation, or surgery [4]. These modern advancements in the treatment of epilepsy have undoubtedly helped patients with epilepsy live a more normal life.

## Conclusions

While the pathophysiology, diagnostics, and treatment have evolved over the last 3000 years, globally, the societal perceptions have largely remained the same. Due to modern medicine and the work of scientists and physicians for millennia, epilepsy can be safely managed, and most patients with the disease can live full and normal lives. There is a direct correlation between society's understanding of epilepsy, and outcomes and wellbeing of patients who have it. Unfortunately, there is still much to be done in regard to the global public perception of the disease, as well as public access to resources.
